# Single Port Laparoscopic Surgery: Concept and Controversies of New Technique

**DOI:** 10.1155/2012/456541

**Published:** 2012-11-11

**Authors:** Boris Kirshtein, Eric M. Haas

**Affiliations:** ^1^Department of Surgery A, Soroka University Medical Center, Beersheba, Israel; ^2^Faculty of Health Sciences, Ben Gurion University of the Negev, P.O. Box 151, Beer Sheva 84101, Israel; ^3^University of Texas Medical School at Houston, Houston, TX 77030, USA

During the last two decades laparoscopic surgery has become the standard care for the management of various emergent and elective surgical pathologies. Well-known advantages of laparoscopy include faster recovery, less postoperative pain, lower perioperative complications, and improved cosmesis. Recent development of laparoscopy was represented with introduction of the concept of scarless surgery using natural orifice transluminal endoscopic surgery (NOTES). Unfortunately, NOTES approach has not yet has been overcome and refused due to need for specialized instruments, learning curve, and prolonged surgery time.

 Single-port laparoscopic surgery (SPLS) is a rapidly evolving technique in the field of minimally invasive surgery. SPLS was initially described in 1992 for gynecologic surgery and in general surgery seven years later. The most commonly reported advantages of SPLS include improved cosmesis, lower morbidity associated with elimination of peripheral ports, shorter length of hospital stay, and potential for decreased incisional pain. Another advantage of this technique is the ability to convert to an alternate minimally invasive procedure such as multiport laparoscopy and avoid the need for an open procedure, therefore maintaining the patient benefits well associated with minimally invasive surgery.

Development of innovative articulated or bent instruments, adjustments in laparoscopes, and developed special multilumen access devices allowed simultaneous multiple instruments insertion facilitated the acceptance of this technique. Increasing application of this modality has been described through case reports and case series proving it is a safe and feasible technique for the surgical treatment of benign and malignant diseases expanded within general, urological, gynecological, and pediatric surgery. Recent publications described using SILS for appendectomy, cholecystectomy, colectomy, adrenalectomy, splenectomy, bariatric surgery, hysterectomy, ovarian cystectomy, and hernias repair. The interest for the technique has gradually progressed toward assessing its efficacy compared with established techniques in minimally invasive surgery. Comparative studies with standard and hand-assisted laparoscopic surgery have shown that SPLS maintains the benefits of minimally invasive surgery. 

There are number of challenges associated with SPLS: special access ports, instrumentation, and surgical techniques. Many of healthcare manufacturers that designed and developed various access devices as SPLS port (Covidien, Mansfield, MA, USA), TriPort (Olympus, Wicklow, Ireland), AirSeal (SurgiQest, Inc., Orange, CT, USA), GelPoint (Applied Medical, Rancho Santa Margarita, CA, USA), Endocone (Karl Stortz GmbH & Co, KG, Tutlingen, Germany) have made single site surgery easier and more efficient. 

 While there exists concern about the learning curve of the experimented surgeons who are used to perform a classic laparoscopic approach, current evidence suggests that this technique can safely be mastered in the hands of experienced laparoscopic surgeons. 

The technical challenges of SILS included limited triangulation and retraction due to confinement of the instrumentation to a single axis, requiring a greater level of surgical experience. A cross-hand technique, flexible tip scope, and articulated instruments have been developed to solve this problem. However, more surgeons reported effective using regular laparoscopic instruments for SPLS procedures. 

 The concept of performing laparoscopic surgery through a single incision is gaining momentum among patients, surgeons, and industry alike. SPLS is a developing field and, to date, level I and II clinical data on the benefits of SILS are lacking. The various publications relating to the technique are mostly case reports or small series describing the feasibility and technical problems of operations. Most of them suggested that SPLS procedures are comparable to the standard laparoscopic surgery. Furthermore, increasing experience of SPLS will continue to innovate to further improve the ergonomics, feasibility, and range of the technique. This special issue included various reports about using SPLS approach in different surgical fields.

Appendectomy is one of the most frequent urgent surgical procedures. Regarding the use of SPLS for appendectomy, a multicentric study comparing SPLS with standard laparoscopic surgery shows the feasibility and success of this technique extended to not only uncomplicated patients but also to complicated and obese patients. There was no difference shown in spite of surgery time, postoperative pain, and postoperative complications. Patients were satisfied with better cosmesis following SPLS appendectomy.

 In regards to biliary tract surgery, laparoscopic cholecystectomy still remains as the gold standard for the surgical removal of the gallbladder in the treatment of symptomatic cholelithiasis. The question is if SPLS cholecystectomy can replace traditional surgical approach. The cosmetic results offered by SPLS have resulted in improved patient satisfaction with the final incision versus the four scars created by the conventional laparoscopic approach. A thorough literature review, technical challenges and instrumentation, complications and outcomes of SPLS cholecystectomy are presented along the work in this special article. in authors' opinion SPLS cholecystectomy is long way off from replacing laparoscopic cholecystectomy due to increased rate of complications with longer operative time, lack of standardization and instrumentation, and needs an additional development. 

 In the field of colon and rectal surgery, the feasibility and the safety of the SPLS colectomy are gaining acceptance for the treatment of colon diseases and colon cancer. This article not only presents the accepted and proposed advantages for this surgical technique but it also makes it clear to us that some disadvantages are yet to be conquered before the procedure can become the standard in minimally invasive colorectal surgery.

 The application of SPLS to a wide variety of procedures is explored during this special edition article as well. A literature review of the use of SPLS in patients with inflammatory bowel disease shows that in experienced hands the use of SPLS is feasible even in patients with complications when used in well-selected patients. The paper showed wide range of possible complications following single incision surgery for IBD and again absence of procedure standardization. 

Following experience in adult surgery pediatric surgeons became SILS in children for appendectomy, cholecystectomy, pyloromyotomy, splenectomy and nephrectomy. In this special edition, we present the evolution of SPLS since the original development until the application into the pediatric field and the benefits of these techniques. Even with the success that exists in pediatrics with the use of these minimally invasive techniques, great obstacles will need to be overcome in order to optimize the approach in children.

Herein, we tried to show controversies in various aspects of single incision surgery. Patients and surgeons enthusiasm, technical progress, and prospective randomized control trials will show the future way and availability of this technique. We hope that you enjoy this special edition as much as we enjoyed working on it.

*Boris Kirshtein* *Boris Kirshtein* *Eric M. Haas* *Eric M. Haas*

